# Development and psychometric properties of Iranian women childbirth experience questionnaire

**DOI:** 10.1002/nop2.752

**Published:** 2020-12-30

**Authors:** Monirolsadate Hosseini Tabaghdehi, Afsaneh Keramat, Zohreh Shahhosseini, Sakineh Kolahdozan, Mahmood Moosazadeh, Zahra Motaghi

**Affiliations:** ^1^ Student Research Committee School of Nursing and Midwifery Shahroud University of Medical Sciences Shahroud Iran; ^2^ School of Nursing and Midwifery Shahroud University of Medical Sciences Shahroud Iran; ^3^ Sexual and Reproductive Health Research Center Mazandaran University of Medical Sciences Sari Iran; ^4^ Department of Medicine School of Medicine Shahroud University of Medical Sciences Shahroud Iran; ^5^ Gastrointestinal Cancer Research Center, Non‐communicable Diseases Institute Mazandaran University of Medical Sciences Sari Iran; ^6^ School of Nursing and Midwifery Shahroud University of Medical Sciences Shahroud Iran

**Keywords:** childbirth, nurses, nursing, psychometric, questionnaire

## Abstract

**Aim:**

This study aimed to develop and psychometrics a questionnaire for assessing childbirth experience in Iranian women.

**Design:**

Cross‐sectional study.

**Methods:**

This cross‐sectional study was done in women who experienced childbirth within the last 12 hr to 2 months from May to December 2018. Questionnaire items were extracted from a comprehensive review of the available studies and questionnaires on childbirth experiences and definitions implied by qualitative interviews. The designed questionnaire was validated in three stages: face, content and construct. Cronbach's alpha was used to determine the reliability of the instrument.

**Result:**

Iranian women childbirth experience questionnaire contained seven factors with 52 items which were called professional support, husband's and other important support, baby, preparation, fear, positive perception and control were extracted. The Cronbach's alpha coefficient after factor analysis was 0.62–0.92 and for the whole instrument was 0.91. The findings showed that Iranian women childbirth experience questionnaire was valid and reliable.

## 1 INTRODUCTION

The experience of childbirth is an individual and important life event for women, which is complex. It is a psychological and physiological process that is influenced by the social, environmental, organizational and policy factors in a society (Larkin et al., 2009).

The varies factors related to childbirth experience including: fear (Elvander et al., 2013; Henriksen et al., 2017), self‐efficacy (Al Ahmar & Tarraf, 2014; Christiaens & Bracke, 2007), participation (Hodnett, 2002; Waldenström, 1999; Waldenström et al., 1996, 2004), control (Al Ahmar & Tarraf, 2014; Bryanton et al., 2008; Christiaens & Bracke, 2007; Goodman et al., 2004; Henriksen et al., 2017; Waldenström, 1999; Waldenström et al., 2004), expectations (Bryanton et al., 2008; Christiaens & Bracke, 2007; Goodman et al., 2004; Waldenström et al., 1996), preparation (Al Ahmar & Tarraf, 2014; Goodman et al., 2004), husband support (Attanasio et al., 2014; Bryanton et al., 2008) and care provider support (Hodnett, 2002; Ulfsdottir et al., 2014; Waldenström, 1999; Waldenström et al., 1996, 2004).

A systematic review study by Hosseini Tabaghdehi et al. (2019) shown that the prevalence of childbirth negative experiences varied from 6.4%–44%, although these assessments were conducted with a different instrument. The childbirth experience affects the individual and the community, so that the negative experience of labour affects the woman's subsequent fertility rate and reduces their subsequent fertility (Gottvall & Waldenström, 2002). On the other hand, positive labour experience can empower women and increase their self‐efficacy and self‐esteem. As a result, it increases women's desire to choose a normal delivery for the next delivery (Hosseini Tabaghdehi et al., 2020).

Considering the decreased fertility rate in Iran within recent years, policymakers in the health system felt the need to take steps to promote the pleasant experiences of childbirth and to encourage vaginal delivery; but such developments require adequate information on the labour experiences of this group of women, and using the proper instruments for diagnosing their labour experiences.

Research‐instrument designers believe that the content of the instrument should be extracted directly from the reference people. Besides, the content of an instrument must be consistent with the culture and lifestyle of the communities or countries where the instruments are supposed to be applied. An instrument designed in a particular country reflects the language and culture of that society and even a precise translation cannot help applying the same content in another country (Shahhosseini et al., 2011). Considering the importance of childbirth experiences and the lack of reliable research instruments in this field in Iran, the present study aimed to design and psychometrics a questionnaire to evaluate different dimensions of childbirth experiences in women with vaginal delivery history.

## 2 MATERIALS & METHODS

This study aimed to provide an instrument to evaluate the childbirth experiences in women with vaginal delivery history.

### 2.1 Research questions

1. Is IWCBEQ a valid questionnaire for evaluating women's labour experiences during labour and delivery?
2. Which factors are effective in the labour experience of women during labour and childbirth?

#### 2.1.1 Setting

This study was conducted in Shahroud University of Medical Sciences and Mazandaran University of Medical Sciences from May–December 2018.

### 2.2 Ethical considerations

The present study was supported by Shahroud University of medical sciences (grant No 97161). The purpose of this study was explained to the participants, and the questionnaire was given after their permission for participation.

### 2.3 Inclusion criteria

Women with uncomplicated vaginal delivery during the last 12 hr up to two months.

### 2.4 Participants

Qualitative interviews were performed on 10 women; the face validity stage included 20 women and construct validity included 781 women who experienced childbirth within the last 12 hr to 2 months. Content validity in the qualitative stage included 10 experts and the quantitative part included 15 experts in the field of reproductive health, midwifery and instrument making.

### 2.5 Instrument

The preparation of the instrument had two stages:

1. Extraction of questions by examining related studies and existing questionnaires on labour experiences and taking qualitative interview from 10 women during 12 hr to 2 months after their vaginal delivery. Qualitative interviews were conducted from May–June 2018 at Abbas Abad health centre.
2. Validity and reliability of the instrument:

Instrument validity consists of 3 stages of face, content and construct validity (Table 1).

**TABLE 1:** Validity and reliability process of questionnaire

Face validity	Impact score for each item	Item with impact score < 1/5 were deleted
Content validity	Item CVR score for each item, Item CVI score for each item	Item with CVR < 0/49 and CVI < 0/79 were deleted
Construct validity	Exploratory factor analysis	‐ KMO > 80 suitability of data for factor analysis ‐ Barlett's test(*p* < 0/005) Eigenvalues > 1 factors should explain 50%–60% of the total variance Factor loading > 0/4
Internal reliability	Cronbach's coefficient	Cronbach's ≥ 0/7 satisfactory

The face validity was performed qualitatively and quantitatively. In the qualitative section, the viewpoints of 20 women with vaginal delivery were used to evaluate they are appropriate, relevant and understandable the items on a questionnaire. In the quantitative section, face validity was measured using Impact score for each item (impact score = importance × frequency (%)) and the questionnaire was given to 20 participants who had vaginal childbirth and the questions with an Impact score of <1.5 were deleted (Mohammadbeigi et al., 2015).

Content validity had two qualitative and quantitative sections. In the qualitative section, the viewpoints of 10 experts on reproductive health and gynaecologist were used. In the quantitative section, the viewpoints of 15 faculty members and professionals with expertise in reproductive health, midwifery and gynaecology were used. In this stage, CVR and CVI were used. CVR (Content Validity Ratio) indicates the necessity of an item. In this study, the minimum amount of CVR was 0.49, according to the Lawshe table, with 15 experts. The CVI (Content Validity Index) determines the relevance and simplicity of each item in the questionnaire. The items with the index of 0.79 or higher were accepted (Polit & Beck, 2006). To determine construct validity, the “exploratory factor analysis” method was used to explore the internal relationship of variables to explore the class of variables, which were often correlated.

The KMO indicates the sampling adequacy index and the Barlett's test and the inflection point 1 was considered indicating the minimum factor load required to maintain each expression in the factors extracted from the factor analysis. Varimax rotation was used to determine the matching and naming of extracted factors (Vakili, 2018).

### 2.6 Instrument reliability

Cronbach's alpha was used to determine the reliability of the instrument. Cronbach's alpha represents the proportion of a group of expressions creating a structure. For good and adequate internal consistency, Cronbach's alpha should be above 0.7 (Mohammadbeigi et al., 2015).

## 3 RESULTS

A total 781 women who were participated for study, 411 (52/8%) women were primipara and 370 (47/4%) were multipara. Table 2 presents characteristic of 781 women with vaginal birth.

**TABLE 2:** Characteristics of the participating women of the sample (*N* = 781)

Variables	*N* (%)	*N* (%)
Age	Women	Husband's
≤20	77 (9/9)	3 (0/4)
21–25	175 (23/4)	66 (8/5)
26–30	287 (36/7)	267 (34/2)
31–35	164 (21)	232 (29/8)
36–40	67 (8/6)	137 (17/5)
≥41	11 (1/4)	75 (9/6)
Total	781 (100)	781 (100)
Educational level		
Under diploma	249 (31/9)	262 (33/5)
Diploma	323 (41/4)	315 (40/3)
University	209 (26/7)	204 (26/2)
Total	751 (100)	781 (100)
Obstetric features		
Gravidity		
1	354 (45/3)	
2	303 (38/8)	
3	80 (10/2)	
≥4	44 (5/7)	
Total	781 (100)	
Parity		
Primipara	413 (52/9)	
Multipara	368 (47/1)	

### 3.1 Validity

Based on an extensive review of the available studies and questionnaires on childbirth experiences and definitions implied by qualitative interviews, a questionnaire containing 148 items on the Likert scale of 5 points (totally agree, agree, no comment, disagree, totally disagree) was designed.

### 3.2 Face validity

The face validity of the prepared questionnaire was carried out both qualitatively and quantitatively. In the qualitative section, the number of items was decreased from 148–133 items. In the quantitative section, the terms with an Impact score of less than 1.5 were deleted and the number of items reached from 133–121.

### 3.3 Content validity

In the content validity section, items with a CVR score of <0.49 and a CVI of less than 0.79 were deleted, so the number of items reached to 74.

### 3.4 Structure validity

To determine the validity of the structure, exploratory factor analysis was carried out in 781 completed questionnaires. The adequacy of the sample was tested using the Kaiser–Meyer–Olkin test, which was 0.91. Then, to determine whether the correlation matrix is significant, Bartlett's test was used to find out whether the factor analysis is justifiable or not; the result was 2,145 (*p* < .001). After calculating the correlation matrix, factors were extracted and the latent factors in the instrument were extracted using principal component analysis and varimax rotation analysis. The inflection point of 0.4 was considered as the minimum load factor required maintaining the expressions in the factors extracted from factor analysis. In initial analysis indicated a 17‑factor structure for the questionnaire, which represented 60.224% of the variance. To simplify and interpret the factor constructs of the questionnaire designed and considering the low power of explaining end factors and considering the degree of consistency of the extracted factors with the concept, by limiting the extraction of factors to 10 factors and using varimax rotation analysis. A total of 10 factors with a special value above one were found which represented 53.553% of the variance. Therefore, 22 items that failed to reach a minimum factor load of 0.4 or had repetitive concepts were removed and 52 items remained. For simplifying and interpretability of the extracted factors with the concepts of labour experiences addressed in this study, the number of factors was reduced to seven factors, which were called professional support, husband's and other important support, baby, preparation, fear, positive perception and control (Table 3). The scoring method was between 1–5; the lowest score for the option of “totally disagree" (score 1) and the highest score for “totally agree” (score 5). The scoring of each structure was determined by calculating the mean scores of items in that structure. The total score of the questionnaire was determined by calculating the mean total score of the whole items. In this questionnaire, the more the total scores of labour experience, the more positive and pleasant the experiences.

**TABLE 3:** Exploratory factor analysis of Iranian women childbirth experience questionnaire (IWCBEQ)

	1	2	3	4	5	6	7	Cronbach's alpha
Eigenvalue	14/353	4/312	3/587	2/630	2/164	1/920	1/641	
Explained variance	21/747	6/533	5/435	3/985	3/279	2/909	2/487	
Cumulative variance	21/747	28/280	33/715	37/699	40/978	43/888	49/043	
**Professional support**								
1. The midwife had a friendly treat with me	0/615	0/040	0/101	0/074	0/333	0/166	0/031	0/92
2. I had a good interaction with my midwife	0/509	0/012	0/117	0/103	0/348	0/236	0/110	
3. The midwife provided the necessary training for control during labour and birth	0/656	0/091	0/238	0/013	0/112	0/093	0/009	
4. The delivery room staff had a good relationship with me during labour and birth	0/759	0/007	0/122	0/131	0/051	0/036	0/018	
5. Midwife understood my needs and wishes	0/817	0/089	0/129	0/029	0/118	0/020	0/103	
6. The midwife treated with me polite and respectful	0/812	0/033	0/106	0/050	0/056	0/011	0/026	
7. The midwife spent enough time for me	0/818	0/079	0/029	0/139	0/047	0/067	0/027	
8. My midwife was aware of what was happening during childbirth	0/756	0/089	0/057	0/088	0/078	0/005	0/153	
9. The midwife cared for me well	0/770	0/95	0/028	0/158	0/059	0/104	0/075	
10. The delivery room midwife had a calm mood	0/683	0/29	0/011	0/137	0/072	0/232	0/015	
11. The midwife encouraged me to adapt to childbirth and continue the process	0/661	0/075	0/119	0/168	0/071	0/097	0/105	
12. My wishes for delivery by midwives were taken into consideration	0/540	0/154	0/211	0/075	0/130	0/209	0/225	
13. The delivery environment was safe and comfortable	0/495	0/074	0/054	0/300	0/118	0/183	0/140	
14. When entering the delivery ward has been warmly welcomed me	0/507	0/054	0/036	0/218	0/082	0/128	0/116	
15. I received the appropriate, on time and necessary services in the delivery ward	0/432	0/004	0/004	0/313	0/040	0/301	0/020	
**Preparation**								
16. I thought that I had the ability and power of natural childbirth	0/171	0/621	0/032	0/125	0/019	0/325	0/058	0/72
17. I liked to experience natural birth	0/046	0/748	0/052	8	0/062	0/241	0/028	
18. The natural birth pain is ultimately sweet	0/050	0/712	0/151	0/083	0/043	0/096	0/080	
19. I planned to get pregnant at the appropriate time	0/074	0/447	0/300	0/017	0/060	0/187	0/011	
20. I felt happy during my childbirth	0/084	0/450	0/081	0/297	0/186	0/130	0/399	
21. I was very hopping during the labour and birth	0/165	0/472	0/103	0/366	0/065	0/044	0/204	
22. I was familiar with delivery environment before birth	0/125	0/041	0/265	0/137	0/076	0/198	0/561	
**Baby**								
23. I was very eager to see the baby during labour	0/108	0/185	0/591	0/039	0/140	0/086	0/186	0/77
24. Immediately after childbirth, I was encouraged to embrace the baby	0/132	0/112	0/738	0/033	0/140	0/052	0/074	
25. Immediately after childbirth, I heard the cry of my baby	0/096	0/078	0/675	0/080	0/053	0/049	0/046	
26. Immediately after childbirth, I was able to see my baby for the first time in a satisfactory method	0/171	0/043	0/685	0/119	0/154	0/032	0/087	
27. I kept my baby for the first time as I wanted	0/129	0/011	0/633	0/176	0/084	0/174	0/197	
**Positive perception**								
28. I had a healthy and safe childbirth	0/232	0/104	0/204	0/415	0/336	0/183	0/009	0/75
29. I have good memories of childbirth	0/064	0/571	0/026	0/145	0/135	0/246	0/250	
30. I felt more responsible, after giving birth	0/101	0/306	0/437	0/409	0/006	0/172	0/115	
31. I thought the childbirth was very painful	0/068	0/660	0/123	0/185	0/016	0/018	0/313	
32. After giving birth, I felt light and comfortable	0/260	0/056	0/181	0/586	0/123	0/019	0/036	
33. *After delivery, I understand my inner strength*	0/078	0/163	0/058	0/225	0/026	0/024	0/007	
34. I felt empowered after childbirth	0/190	0/080	0/028	0/696	0/031	0/169	0/063	
35. I felt successful with childbirth	0/188	0/193	0/171	0/684	0/017	0/185	0/087	
36. I had the feeling of independence and self‐sufficiency with childbirth	0/107	0/235	0/208	0/603	0/105	0/066	0/018	
37. My self‐confidence increased with childbirth	0/167	0/154	0/224	0/519	0/152	0/019	0/195	
**Husband's and other important support**								
38. The support and presence of my parents helped during the labour	0/114	0/073	0/178	0/101	0/594	0/144	0/099	0/77
39. The presence of my husband's strengthen my heart	0/106	0/059	0/179	0/053	0/738	0/058	0/044	
40. My husband's support during the labour was helpful	0/126	0/025	0/132	0/072	0/765	0/076	0/124	
41. During my pregnancy, my husband's encouraged me to have a natural birth	0/172	0/069	0/034	0/001	0/657	0/110	0/169	
42. During my pregnancy, my family encouraged me to have a natural birth	0/153	0/090	0/091	0/114	0/559	0/084	0/147	
**Control**								
43. I believed that after natural childbirth, I have the ability to do my baby's work alone	0/116	0/205	0/054	0/173	0/082	0/515	0/020	0/74
44. I could participate in decision‐making of labour and childbirth	0/226	0/250	0/098	0/065	0/126	0/484	0/235	
45. I have gained knowledge about childbirth using various resources	0/034	0/214	0/175	0/018	0/115	0/624	0/069	
46. I was ready for my childbirth with the knowledge of how labour pain and methods of dealing to childbirth.	0/189	0/234	0/132	0/043	0/128	0/626	0/216	
47. I tolerated the pain of labour more effectively with Relying on good thoughts	0/152	0/332	0/003	0/171	0/188	0/602	0/090	
48. When I was talking to my midwife, I was able to tolerate labour pain	0/354	0/209	0/019	0/153	0/151	0/525	0/010	
49. *The crowd of the environment* reduced my tolerance	0/049	0/133	0/025	0/054	0/056	0/063	0/652	
**Fear**								
50. I was afraid of hurting and dying	0/009	0/116	0/054	0/024	0/079	0/230	0/636	0/62
51. I was afraid of labour pain	0/007	0/082	0/039	0/021	0/016	0/018	0/721	
52. I was worried and anxious during labour and childbirth	0/044	0/015	0/046	0/056	0/148	0/003	0/616	

The results of the reliability of the questionnaire before the factor analysis indicated the Cronbach's alpha coefficient of 0.94. Also, the Cronbach's alpha coefficient after factor analysis was 0.62–0.92 and for the whole instrument was 0.91 (Table 3).

## 4 DISCUSSION

The purpose of this study was to design an instrument for assessing the labour experience of women with vaginal delivery. The framework of this tool is based on an extensive review of available studies and questionnaires on childbirth experiences and qualitative interviews with women who had vaginal delivery within the last 12 hr to 2 months. Then, the validity and reliability of the tool were done. Its validity consists of three stages (face, content and construct) and its reliability was Cronbach's alpha. To determine the face validity of the 20 contributors used, there are similar studies that have been used by the participants to examine face validity.

Content validity has been used qualitatively and quantitatively using experts' opinions. They have used this approach in several studies to determine the validity of their tools (Gungor & Beji, 2012; Moghaddam‐Banaem et al., 2017). To determine the validity of the constructor, the researcher has used exploratory factor analysis, which IWCBEQ questionnaire consists of 52 items which were categorized into seven factors called professional support, husband's and other important support, baby, preparation, fear, positive perception and control.

### 4.1 Professional support

The first factor with a special value of 14.35 at 21.78% had the highest contribution in explaining the total variance. Some other studies also considered professional support as an important factor for labour experience and delivery satisfaction (Carquillat et al., 2017; Dencker et al., 2010; Ford et al., 2009; Martin & Fleming, 2011; Sjetne et al., 2015; Smith, 2001) which indicates the importance of the role of Midwife in creating a pleasant childbirth experience.

Professional support makes women feel they have seen and feel safe and secure (Dahlberg et al., 2016). This support includes presence, accountability and confidence. Professional support makes empowerment for women to have positive childbirth experience (Nilsson et al., 2013).

### 4.2 Preparation

The second factor with a high variance in the exploratory factor analysis was the preparation with a special value of 4.31, explaining about 6.53% of the total variance. The study Aune et al determined that planned pregnancy and couple's readiness has a significant role in promoting positive childbirth experiences (Aune et al., 2015) Also, in the study of Karlstrom et al., the acquisition of knowledge about delivery and its process leads to mental and physical preparedness in women which creates realistic expectations in them, thus fulfilling these realistic expectations has an important role in promoting childbirth experiences (Karlström et al., 2015). In this study, preparation for evaluating the labour experience and delivery satisfaction was studied as factors affecting maternity satisfaction.

### 4.3 Baby

In this study, early and satisfactory child contact was the third important determinant of labour satisfaction, with a special value of 3.59 explaining about 5.44% of the total variance. In line with this study, a systematic review conducted by Hosseini Tabaghdehi et al. (2019) showed that one of the factors affecting labour experience is the role of the child. In a qualitative study, the mothers defined positive experience as a healthy child (Hardin & Buckner, 2004) The child's role was evaluated in labour satisfaction instrument (Carquillat et al., 2017; Martin & Fleming, 2011).

### 4.4 Positive perception

The fourth factor in this instrument was the positive perception of labour, with the special value of 2.63% and 3.99% of the total variance. In the qualitative section, women mentioned empowerment, accountability, self‐efficacy and independence as part of their delivery experience (Shahoei et al., 2014). There are a variety of instruments which considered perception of labour and childbirth to evaluate of delivery experience (Carquillat et al., 2017; Dencker et al., 2010; Martin & Fleming, 2011; Smith, 2001; Truijens et al., 2014).

Another qualitative study conducted by Nilsson et al. (2013) showed that empowerment is an opportunity to promote the childbirth experience. In their study, physical confidence, the interaction between the mind and body and the continued support had an important role in the empowerment of women.

*The husband's and other import support* was the fifth factor with the special value of 2.16 explaining about 3.28% of the total variance. Inconsistent with the present study, there are other instruments which considered the role of husband and relatives in the labour experience (Carquillat et al., 2017; Martin & Fleming, 2011; Smith, 2001).

### 4.5 Control

The sixth factor of this tool was control, which, with a value of 1.92, accounts for about 2.91% of the total variance. In the study of Nilsson et al. (2013) the interaction between mind and body is considered as a kind of control. According to the studies, control includes internal and external control:

- *Internal control* includes thoughts, behaviour, pain and physical function,
- *external control* including the pains, information, environment, decisions and procedures and the outcome of delivery (Colley et al., 2018). In the factor analysis, thoughts, behaviour, physical performance, information, environment and decisions were maintained with high factor load.

### 4.6 Fear

The seventh factors were fear with the special value of 1.64 explaining 2.49 of the total variance. In the studies has been determined that fear influencing on the negative experiences and also led to the choice of caesarean section for the next delivery (Al Ahmar & Tarraf, 2014; Christiaens & Bracke, 2007).

The eighth and tenth factors had low Cronbach's alpha coefficient and the ninth factor had limited number of items (two items); so by confirming the research team, they were merged with other factors given the importance of their interpretability. The eighth factor included question 22: “I was familiar with the birth environment before giving birth,” with a factor load of 0.56 merged with the factor of preparation and question 49: “The crowd of the environment reduced my tolerance.” with a factor load of 0.652 merged with the control factor. Questions 22 and 49 show the role of the delivery environment in the labour experience. In the studies were found that providing a suitable environment and familiarity with the delivery environment have a significant role in the progress of delivery (Askari et al., 2010; Bayrami et al., 2011). Because the proper environment makes women feel secure. According to the study by Judith et al, a sense of safety during labour can reduce catecholamine levels and, as a result, labour progress (Lothian, 2004). The ninth factor included the questions: “I tolerated the pain of labour more effectively with relying on good thoughts” with a factor load of 0.95 merged with the control factor; “After delivery, I understand my inner strength” with a factor load of 0.90 merged with the factor of positive perception. The 10th factor includes questions: “When entering the delivery ward has been warmly welcomed me” with a factor load of 0.559; “I received the appropriate, on time and necessary services in the delivery ward” with a factor load of 0.47 which were merged with the professional support factor. In this questionnaire, questions 29 and 31 were included in the second factor (preparation) and, respectively, had a gain of 0.77 and 0.66 which, according to the research team, were considered as a positive perception factor.

The reliability is one of the most important criteria that show the quality of the research instrument. The labour experience Questionnaire has had an internal consistency and acceptable sustainability, so that the Alpha Cronbach's after factor analysis was 0.62–0.9 for the factors of the questionnaire and 0.91 for the whole instrument, indicating the internal consistency of the domains and questions (Table 3).

The strengths of this study are:

1. Extracting items by reviewing the available studies and questionnaires in this field, face‐to‐face interviews with women who have experienced vaginal delivery.
2. Determining the construct validity using a big sample size (781 samples).
3. Labour experiences of women in this study were analysed 12 hr to two months after their delivery, which is not a long time interval to play an intervention role.

## 5 LIMITATIONS

The limitation of this study is the lack of examining women's behavioural status before and during pregnancy, since it has a significant negative effect on the perception of labour.

## 6 CONCLUSION

Based on the results of this study, Iranian women childbirth experience questionnaire was valid and reliable. IWCBEQ questionnaire can be used to identify negative childbirth experience, planning, intervention and promoting healthcare services. Therefore, it is recommended to use this questionnaire in other studies.

## CONFLICT OF INTEREST

The authors have no conflicts of interest relevant to this article.

## Data Availability

The data that support the findings of this study are available from the corresponding author, upon reasonable request.
